# Calcium-permeable ion channels in control of autophagy and cancer

**DOI:** 10.3389/fphys.2013.00272

**Published:** 2013-10-02

**Authors:** Artem Kondratskyi, Maya Yassine, Kateryna Kondratska, Roman Skryma, Christian Slomianny, Natalia Prevarskaya

**Affiliations:** Laboratory of Excellence, Equipe Labellisée par la Ligue Nationale Contre le Cancer, Ion Channels Science and Therapeutics, INSERM, U-1003, Université Lille 1Villeneuve d'Ascq, France

**Keywords:** calcium, autophagy, TRP, ion channels, cancer

## Abstract

Autophagy, or cellular self-eating, is a tightly regulated cellular pathway the main purpose of which is lysosomal degradation and subsequent recycling of cytoplasmic material to maintain normal cellular homeostasis. Defects in autophagy are linked to a variety of pathological states, including cancer. Cancer is the disease associated with abnormal tissue growth following an alteration in such fundamental cellular processes as apoptosis, proliferation, differentiation, migration and autophagy. The role of autophagy in cancer is complex, as it can promote both tumor prevention and survival/treatment resistance. It's now clear that modulation of autophagy has a great potential in cancer diagnosis and treatment. Recent findings identified intracellular calcium as an important regulator of both basal and induced autophagy. Calcium is a ubiquitous secondary messenger which regulates plethora of physiological and pathological processes such as aging, neurodegeneration and cancer. The role of calcium and calcium-permeable channels in cancer is well-established, whereas the information about molecular nature of channels regulating autophagy and the mechanisms of this regulation is still limited. Here we review existing mechanisms of autophagy regulation by calcium and calcium-permeable ion channels. Furthermore, we will also discuss some calcium-permeable channels as the potential new candidates for autophagy regulation. Finally we will propose the possible link between calcium permeable channels, autophagy and cancer progression and therapeutic response.

## Autophagy

Autophagy is a cellular catabolic process for the degradation and recycling of protein aggregates, long-lived proteins and damaged organelles to maintain cellular homeostasis (Ravikumar et al., 2010; Chen and Klionsky, 2011). Normally, autophagy occurs under basal conditions but it can be stimulated in response to different types of cellular stress, such as nutrient starvation, hypoxia, endoplasmic reticulum (ER) stress, oxidative stress, mitochondrial damage as well as treatment with some pharmacological agents (Kroemer et al., 2010). To date, three types of autophagy have been described, including macroautophagy, microautophagy, and chaperone-mediated autophagy (CMA) (Klionsky, 2005). These types differ in their functions and regulatory mechanisms. During macroautophagy, further mentioned as autophagy, cytoplasmic components are engulfed by double membrane structures termed autophagosomes that mature by fusing first with late endosomes or directly with lysosomes to form autolysosomes. Finally, the content of autolysosome together with its inner membrane is degraded by lysosomal hydrolases to be reused in the cell (Ravikumar et al., 2010; Chen and Klionsky, 2011).

The process of autophagosome formation consists of several stages, namely initiation, elongation and maturation and fusion. At present, around 30 autophagy-related genes are identified, and most of them have mammalian orthologs (Ravikumar et al., 2010). These genes are implicated in different stages of autophagy. Thus, the autophagosome formation requires the activity of the class III phosphatidylinositol 3-kinase (PI3K), Vps34. Mammalian Atg18 homolog WIPI-1 binds to PI3P, the product of Vps34 activity, and is recruited to autophagosomal membrane. Vps34 is a part of a protein complex containing Beclin1/Atg6, p150/Vps15 and Atg14/barkor proteins (He and Levine, 2010). Another complex implicated in the initiation stage of autophagosome formation is the focal adhesion kinase family interacting protein FIP200-ULK1/2/Atg1-Atg13 complex, where Atg13, ULK1, and ULK2 proteins have been shown to be direct targets of the mammalian target of rapamycin (mTOR) (Mizushima, 2010). Under nutrient-rich conditions, mTOR is associated with this complex, whereas under starvation conditions inhibition of mTOR causes its dissociation from the complex and leads to activation of ULK1/2, subsequent phosphorylation of FIP200 and autophagy induction (Mizushima, 2010; Kim et al., 2011). The elongation stage requires cleavage of the microtubule-associated protein 1 light chain 3 (Atg8/LC3) by Atg4, resulting in the formation of cytosolic LC3-I protein, which is conjugated to phosphatidylethanolamine (PE) to form membrane bound LC3-II (Tanida et al., 2004). Another stage is the formation of Atg5–Atg12–Atg16L1 protein complex which facilitates LC3-I conjugation to PE and determines the sites of LC3 lipidation. Thus, LC3-II is specifically targeted to the autophagosome membrane and remains associated with autophagosome even after fusion with the lysosomes (Geng and Klionsky, 2008; Ravikumar et al., 2010). This peculiarity makes it a useful marker for autophagy research (Klionsky et al., 2012).

## Autophagy in cancer

Autophagy is thought to be predominantly a cell-survival mechanism. Under basal conditions, autophagy helps to maintain cellular homeostasis through the elimination of damaged organelles and protein aggregates, whereas in stress autophagy provides nutrients from macromolecules, produces energy, removes potentially dangerous elements thus assures cytoprotective response to support cell survival (Mizushima et al., 2008; Ravikumar et al., 2010). Deregulation of autophagy is known to affect many physiological processes and has been implicated in a number of diseases, such as neurodegenerative disorders and cancer (Choi et al., 2013). In cancer, current evidence indicate that autophagy may act as a tumor suppressor, in particular at the early stages of tumor initiation (White, 2012). Indeed, a number of autophagy related proteins, including Beclin1 (Liang et al., 1999; Yue et al., 2003), ATG5 (Yousefi et al., 2006; Takamura et al., 2011), ATG4c (Marino et al., 2007), and ATG7 (Takamura et al., 2011) as well as some accessory proteins (Bif1, UVRAG) (Liang et al., 2007a; Takahashi et al., 2007) have been shown to be tumor suppressors. In addition, several oncosuppressor proteins, such as DAPK (Inbal et al., 2002), PTEN (Arico et al., 2001), TSC1/2, p53 (Feng et al., 2005) and LKB1 (Liang et al., 2007b) have been recently shown to stimulate autophagic process, while a number of oncoproteins, like Bcl-2 (Pattingre et al., 2005), AKT and class I PI3K (Arico et al., 2001) negatively regulate autophagy (Rubinsztein et al., 2012). One of the possible mechanisms for antitumorigenic functions of autophagy is based on its cytoprotective role. More specifically, inhibition of autophagy will favor DNA damage, genomic instability and thus tumorigenesis through the accumulation of protein aggregates, damaged mitochondria and reactive oxygen species (ROS) (White, 2012).

In contrast, in established tumors, autophagy being a primarily survival mechanism can support cancer cell survival in harsh conditions, characterized by nutrient deficiency and hypoxia (Degenhardt et al., 2006; White, 2012). Indeed, accumulated data suggest that autophagy inhibition by genetic or chemical means facilitates apoptotic cell death and inhibits tumor cells growth in several cancers (Amaravadi et al., 2007; Takamura et al., 2011; Yang et al., 2011a, b). In addition, elevated autophagy is often detected in cancer cells in response to radiation and chemotherapy (Ito et al., 2005; Kondo et al., 2005; White, 2012). Furthermore, autophagy seems to contribute to the therapeutic resistance of some cancers, as inhibition of autophagy has been shown to sensitize tumor cells to chemotherapy treatments (Guo et al., 2012; Selvakumaran et al., 2013). Therefore, autophagy inhibition as an adjuvant to chemotherapy represents a promising strategy in the treatment of some cancers (Amaravadi et al., 2011; White, 2012). Indeed, more than 20 clinical trials are currently evaluating the efficacy of chloroquine and hydroxychloroquine (autophagy inhibitors) in treatment of different cancers either in monotherapy or in combination with other anticancer agents. The preliminary results of many of these trials show apparent antitumour activity (Yang et al., 2011b; Kimura et al., 2013). In addition to chloroquine and hydroxychloroquine another autophagy inhibitors, including 3-Methyladenine, bafilomycin A1 and pepstatin A have been shown to enhance the antitumour efficacy of chemotherapeutic drugs both *in vitro* and *in vivo* (Hsu et al., 2009; Li et al., 2010; Cheong et al., 2012; Lamoureux and Zoubeidi, 2013). However, it should be noted that all these autophagy inhibitors are not specific and can modulate other cellular processes, such as endocytosis, lysosomal function etc. Hence unexpected side effects could occur when treating patients with these drugs. Therefore, more specific and potent autophagy inhibitors are clearly needed.

Thus, figuring out whether to stimulate or inhibit autophagy in each particular case will provide a powerful approach to treat cancer.

## Calcium, Ca^2+^-permeable ion channels and cancer

Changes in the cytosolic free Ca^2+^ concentration play a central role in many fundamental cellular processes including muscle contraction, transmitter release, cell proliferation, differentiation, gene transcription and cell death (Berridge et al., 2000). Giving that Ca^2+^ controls so many vital processes, disturbance of the Ca^2+^ homeostasis regulatory mechanisms leads to a vast variety of severe pathologies, including cancer. Indeed, the role of Ca^2+^ is well-established in many cell signaling pathways involved in carcinogenesis (Monteith et al., 2007, 2012; Prevarskaya et al., 2011).

Increase in cytosolic calcium can occur as a result of Ca^2+^ influx from the extracellular space and Ca^2+^ release from intracellular sources. Both Ca^2+^ influx and Ca^2+^ release are tightly controlled by numerous regulatory systems that provide the specific spatial and temporal characteristics of an intracellular calcium signal that are required for sustaining certain cellular functions (Berridge et al., 2000).

Mitochondrial, ER, lysosomal and cytosolic calcium levels are regulated by calcium permeable ion channels localized either on the membranes of the intracellular organelles or on the plasma membrane (Berridge et al., 2003; Rizzuto et al., 2012). The calcium permeable channels, including families of transient receptor potential (TRP) channels, store-operated channels (SOCs), voltage-gated calcium channels, two-pore channels, mitochondrial permeability transition pore (MPTP), mitochondrial calcium uniporter (MCU), IP3 and ryanodine receptors and others contribute to changes in [Ca^2+^]_i_ by providing Ca^2+^ entry pathways, by modulating the driving force for the Ca^2+^ entry, and also by providing intracellular pathways for Ca^2+^ uptake/release into/from cellular organelles (Berridge et al., 2003; Pedersen et al., 2005; Bernardi and von Stockum, 2012; Rizzuto et al., 2012).

Thus, modulation of calcium permeable ion channel's expression/function affects intracellular Ca^2+^ concentrations and consequently calcium dependent processes, such as proliferation, apoptosis and autophagy (Flourakis and Prevarskaya, 2009; Decuypere et al., 2011a; Dubois et al., 2013). Indeed, defects in Ca^2+^ channels expression/function are involved in a number of pathologies, including tumorigenesis, since increased expression of Ca^2+^ channels could lead to elevated cytosolic Ca^2+^ levels and promotion of Ca^2+^-dependent proliferative pathways (Nilius, 2007; Prevarskaya et al., 2010). As an example, several members of the TRP family of ion channels, namely TRPC1, TRPC3, TRPC6, TRPV1, TRPV6, TRPM1, TRPM4, TRPM5, TRPM7, and TRPM8, show altered expression in cancer cells (Shapovalov et al., 2011). The involvement of SOCs, MPTP, MCU, IP3 receptors and ryanodine receptors in the regulation of cell death has also been described (Hajnoczky et al., 2000; Boehning et al., 2004; Flourakis et al., 2010; Wong et al., 2012b; Bernardi, 2013; Curry et al., 2013; Dubois et al., 2013; Qiu et al., 2013).

## Role of Ca^2+^ in autophagy

Recent findings identified intracellular calcium as a key regulator of both basal (Cardenas et al., 2010) and induced (Hoyer-Hansen et al., 2007) autophagy. The complex role for Ca^2+^ in autophagy regulation has become obvious since 1993, when the first report linking autophagy and intracellularly sequestered calcium was published (Gordon et al., 1993). Indeed, Gordon et al. demonstrated that decrease as well as increase in cytosolic Ca^2+^ levels inhibited autophagy in rat hepatocytes (Gordon et al., 1993). And till now, the data on the mechanisms by which calcium controls autophagy remain rather controversial. Several groups reported inhibitory actions of calcium on autophagy, while another proposed mechanisms for calcium to activate autophagy (Decuypere et al., 2011a; Cardenas and Foskett, 2012; Parys et al., 2012). Indeed, Hoyer-Hansen and colleagues provided evidence that a rise in the free cytosolic calcium is a potent inducer of macroautophagy (Hoyer-Hansen et al., 2007). They demonstrated that Ca^2+^ mobilizing agents, namely vitamin D3, thapsigargin, ATP and ionomycin, stimulate autophagy via a signaling pathway involving Ca^2+^ -activated kinase CAMKK-beta, which directly activates AMPK to inhibit mTOR (Hoyer-Hansen et al., 2007). Recently, this pathway was shown to be required for amyloid-beta peptide induced autophagosome formation (Son et al., 2012). Ca^2+^/CAMKK-beta/AMPK pathway, although mTOR-independent, has been found to be involved in the leucine-rich repeat kinase-2 (LRRK2) induced autophagy (Gomez-Suaga et al., 2012). Authors proposed the mechanism in which LRRK2 activates NAADP receptors, in particular TPC2, leading to Ca^2+^ mobilization from acidic stores that in turn stimulates Ca^2+^-induced Ca^2+^ release from ER and subsequent CAMKK-beta/AMPK pathway activation (Gomez-Suaga et al., 2012). In all the cases discussed above, buffering of cytosolic Ca^2+^ with BAPTA-AM effectively inhibited autophagosomes accumulation, confirming the role of Ca^2+^. Another evidence supporting stimulatory effect of Ca^2+^ on autophagy is that exogenously introduced calcium in the form of calcium phosphate precipitates induces macroautophagy, which is Beclin1, Atg5 and PI3K class III dependent (Gao et al., 2008). This effect could be antagonized by extra- or intra-cellular calcium chelation.

In line with activatory role of calcium in autophagy, Sakaki et al. showed that Ca^2+^-dependent activation of protein kinase Ctheta is required for ER-stress induced autophagy but not for starvation induced autophagy (Sakaki et al., 2008).

In another studies calcium/calmodulin dependent death associated protein kinase (DAPK) was shown to positively regulate autophagy in a Beclin1 dependent manner. Thus, DAPK phosphorylates Beclin1, thereby promoting its dissociation from Bcl-X_L_ and Bcl-2 inhibitory proteins (Zalckvar et al., 2009a, b).

On the contrary, Khan et al suggested that basal autophagic flux may be negatively regulated by IP3R-dependent Ca^2+^ release from the ER (Khan and Joseph, 2010). The authors proposed a mechanism in which cytosolic Ca^2+^ elevation acts to maintain an elevated mTORC1 activity through AMPK independent pathway (Khan and Joseph, 2010). Furthermore, amino acids were shown to induce an increase in [Ca^2+^]_i_, supposedly through the influx of extracellular Ca^2+^, which acts to enhance the binding of Ca^2+^/CaM to hVps34, resulting in mTOR activation (Gulati et al., 2008). This pathway could also lead to autophagy inhibition.

Thus, calcium is likely to have different regulatory effects on autophagy, depending on spatial and temporal parameters of Ca^2+^ signaling, nutrient and growth factor availability, as well as pathology (cancer, neurodegenerative disorders, inflammation etc.) (Decuypere et al., 2011a).

## Calcium permeable channels in the control of autophagy

Modulation of calcium permeable channels expression/function affects intracellular Ca^2+^ concentrations and, consequently calcium dependent processes, such as proliferation, apoptosis and autophagy. The role of calcium-permeable channels for proliferation and apoptosis is largely recognized (Monteith et al., 2007; Flourakis and Prevarskaya, 2009; Prevarskaya et al., 2010; Dubois et al., 2013), whereas the information about molecular nature of channels regulating autophagy and the mechanisms of this regulation is still limited.

Hereafter, we will provide an overview of the literature on this subject and discuss the possible involvement of calcium permeable ion channels in the regulation of autophagy.

Most reports considering calcium permeable channels as autophagy regulators focused on the inositol trisphosphate receptor (IP3R), the main intracellular Ca^2+^ release channel (Parys et al., 2012). Together these reports suggested a complex role for IP3R, since both stimulatory as well as inhibitory functions for IP3R toward autophagy have been described.

Thus, in one paper it was suggested that cadmium (Cd^2+^) induces autophagy through elevation of cytosolic calcium via IP3R and subsequent extracellular signal-regulated kinase (ERK) activation (Wang et al., 2008). As a proof for this, the authors showed that 2-aminoethoxydiphenil borate (2-APB), a blocker of IP3R, suppressed, while knockdown of calcineurin, a putative IP3R inhibitor, increased Cd^2+^-induced autophagy (Wang et al., 2008). However, 2-APB was shown to modulate a number of TRP channels, SERCA pump and SOCs (Peppiatt et al., 2003; Clapham, 2007) and as to calcineurin, its role in the IP3R regulation is debated at present (Bultynck et al., 2003).

In another study, IP3R was shown to be required for differentiation factor DIF-induced autophagic cell death in *Dictyostelium discoideum* (Lam et al., 2008). Through random insertional mutagenesis, the authors showed that inactivation of the *iplA* gene, the only gene encoding an IP3R in this organism, prevented autophagic cell death (Lam et al., 2008).

The stimulatory role of IP3R on starvation-induced autophagy has been recently shown (Decuypere et al., 2011b). The authors showed that the Ca^2+^ chelator BAPTA-AM as well as the IP3R inhibitor xestospongin B abolished starvation induced increase in LC3 lipidation and GFP-LC3-puncta formation. Moreover, starvation lead to IP3R sensitization through increased Beclin1 binding to the IP3R (Decuypere et al., 2011b).

To date, most reports on IP3R-dependent regulation of autophagy suggest inhibitory role for IP3R toward autophagy (Parys et al., 2012).

Thus, lithium (Li^+^) was found to induce mTOR independent autophagy through inhibition of inositol monophosphatase and further decrease in IP3 levels (Sarkar et al., 2005). Consistently, another study demonstrated that IP3R inhibitor xestospongin (XeB) or IP3R knockdown induced autophagy in HeLa cells (Criollo et al., 2007). One of the mechanisms, by which XeB and starvation induce autophagy was proposed by Vicencio et al. (2009). The authors suggested that xestospongin B and nutrient starvation disrupt a molecular complex formed by the IP3R, Beclin 1 and Bcl-2, and presented evidence that the IP3R represses autophagy through Bcl-2-mediated binding of Beclin 1, thus suggesting Ca^2+^-independent mechanism (Vicencio et al., 2009).

As three IP3R isoforms exist, some groups studied the impact of IP3R on autophagy in the triple IP3R-deficient DT40 cells (Cardenas et al., 2010; Khan and Joseph, 2010). These cells demonstrate higher basal autophagy levels, compared to wild-type. Interestingly, expression of IP3R3, but not of ryanodine receptor type 2, rescued elevated autophagy in these cells (Cardenas et al., 2010). In contrast, expression of Ca^2+^ impermeable mutant D2550A-IP3R3 failed to suppress constitutive autophagy, suggesting a necessity of the Ca^2+^-release activity for IP3R. The authors proposed the mechanism in which constitutive IP3R mediated Ca^2+^ release and uptake of this Ca^2+^ by mitochondria is fundamentally required to maintain mitochondrial bioenergetics and ATP production in resting cells thereby suppressing autophagy. Absence of this Ca^2+^ transfer results in inhibition of pyruvate dehydrogenase and activation of AMPK, which activates prosurvival macroautophagy in mTOR independent manner (Cardenas et al., 2010).

Along with IP3R some other calcium permeable channels were shown to be involved in autophagy regulation. Among them, TRPML1, also known as mucolipin-1, ubiquitously expressed TRP channel primarily localized to the late endosomal and lysosomal compartments (Zeevi et al., 2009; Cheng et al., 2010). Direct patch-clamp of enlarged lysosomes revealed that TRPML1 is a Ca^2+^ permeable channel (Dong et al., 2008). The main physiological function of TRPML1 channel is considered to serve as a late endosomal/lysosomal Ca^2+^ release channel. Loss-of-function mutations in the human TRPML1 gene result in mucolipidosis type IV, a neurodegenerative lysosomal storage disorder characterized by mental retardation and retinal degeneration (Bach, 2001; Altarescu et al., 2002). Fibroblasts from mucolipidosis type IV patients exhibit enlarged vacuoles with accumulated lipids and acid mucopolysacharides, suggesting the role for TRPML1 in trafficking of proteins and lipids (Riedel et al., 1985; Goldin et al., 1999; Slaugenhaupt et al., 1999). Loss of TRPML1 has been shown to be accompanied by impairment in the lysosomal pH, accumulation of autophagosomes and abnormal mitochondria, accumulation and aggregation of p62 and ubiquitin proteins, all of which suggested a defective autophagy (Jennings et al., 2006; Soyombo et al., 2006; Vergarajauregui et al., 2008; Curcio-Morelli et al., 2010). Indeed, several studies have proposed TRPML1 as autophagy regulator (Vergarajauregui et al., 2008; Venugopal et al., 2009; Curcio-Morelli et al., 2010; Wong et al., 2012a; Venkatachalam et al., 2013). Vergarajauregui et al. showed that accumulation of autophagosomes in TRPML1-deficient fibroblasts obtained from mucolipidosis type IV patients was due to increased Beclin-1 dependent autophagosome formation and delayed fusion of autophagosomes with late endosomes/lysosomes. The authors claimed that TRPML1 is necessary for efficient fusion of both autophagosomes and late endosomes with lysosomes although it is not clear if the Ca^2+^-channel function of the TRPML1 is essential here (Vergarajauregui et al., 2008). In another study, group of S. Slaugenhaupt showed that CMA is impaired in mucolipidosis type IV fibroblasts (Venugopal et al., 2009). The authors showed that TRPML1 directly interacts with Hsc70 and Hsp40, members of molecular chaperone complex required for CMA, and hypothesized that this interaction may be required for intralysosomal Hsc70 to facilitate the translocation of CMA substrate proteins across the lysosomal membrane. The authors also speculated that TRPML1 channel activity is required for CMA (Venugopal et al., 2009). In 2010 same group investigated macroautophagy in neurons isolated from cerebellum of TRPML1^−/−^ mouse embryos (Curcio-Morelli et al., 2010). These cells displayed higher levels of basal autophagy markers compared to wild-type ones. In addition, LC3-II clearance was affected in these cells, suggesting impairment of lysosomal function. However, the link between observed defects in autophagy and functionality of TRPML1 as a Ca^2+^ permeable channel is missing. Recently, Wong et al showed that Drosophila TRPML is required for TORC1 activation (Wong et al., 2012a). Authors demonstrated defects in amphisomes/lysosomes fusion and elevated late endosomal/lysosomal Ca^2+^ levels in flies lacking TRPML1. Authors also showed decreased TORC1 activity and increased induction of autophagy in TRPML1^−^ mutants. Moreover, authors suggested that TORC1 regulates the subcellular localization of TRPML1. Thus, this study points out to TRPML1 as a Ca^2+^ channel present in amphisomes which releases luminal Ca^2+^ to facilitate Ca^2+^-dependent fusion of amphisomes with lysosomes (Wong et al., 2012a).

In addition to TRPML1, another member of mucolipin family, TRPML3 has been shown to be involved in autophagy regulation. In contrast to TRPML1, TRPML3 exhibits more restrictive tissue distribution, and is primarily localized to early as well as late endosomes/lysosomes and less to the plasma membrane (Zeevi et al., 2009; Cheng et al., 2010).

It has been shown that overexpression of TRPML3 leads to increased autophagy in HeLa cells (Kim et al., 2009). Moreover, TRPML3 is recruited to autophagosomes upon induction of autophagy. Additionally, expression of dominant negative mutant TRPML3 (D458K) or knockdown of endogenous TRPML3 by siRNA reduces autophagy. Thus, it has been proposed that TRPML3 provides Ca^2+^ that is required for fusion and fission events in autophagy (Kim et al., 2009). Further, heteromultimerization of TRPML channels was shown to affect autophagy (Zeevi et al., 2010).

Also TRPV1 was proposed to regulate autophagy in thymocytes (Farfariello et al., 2012). The authors showed that capsaicin, an activator of TRPV1, induce Beclin-1 dependent accumulation of LC3-II protein. This effect can be antagonized by capsazepine, a blocker of TRPV1 and compound C, an AMPK inhibitor, suggesting AMPK involvement. The authors proposed that capsaicin induced autophagy is calcium dependent, as cotreatment with EDTA markedly reduced LC3-II accumulation. Moreover, it was shown that capsaicin induces accumulation of ATG4C and triggers its oxidation in a ROS-dependent manner, thus regulating LC3 lipidation levels (Farfariello et al., 2012). However, capsaicin was shown to have TRPV1-independent effects, such as inhibition of voltage-gated calcium channels (Hagenacker et al., 2005), cancer cell growth inhibition and apoptosis induction (Mori et al., 2006; Chow et al., 2007). Additionally, upon prolonged exposure to capsaicin, TRPV1 desensitization occurs and its activity decreases (Caterina et al., 1997). Thus, additional experiments using more specific agonists and antagonists as well as siRNA knockdown are needed to confirm the role of TRPV1 in autophagy regulation. It would be interesting as well to compare the effect of capsaicin on autophagy in TRPV1-expressing and TRPV1-null cells.

Some ion channels, which do not belong to the family of TRP channels, were also proposed to regulate autophagy. Williams et al. found that L-type calcium channels antagonists, namely verapamil, loperamide, nimodipine, nitrendipine and amiodarone induce mTOR-independent autophagy (Williams et al., 2008). Conversely, the L-type Ca^2+^ channel agonist (±)-BAY K 8644 that increases cytosolic Ca^2+^ levels, inhibits autophagy. Authors demonstrated that elevated cytosolic Ca^2+^, presumably due to activity of L-type calcium channels on the plasma membrane, can activate calpains, a family of Ca^2+^-dependent cysteine proteases, which cleave and activate the α-subunit of heterotrimeric G proteins G_s_α. G_s_α activation, in turn, increases adenylyl cyclase activity leading to increase in cAMP levels. Next, elevated intracellular cAMP levels negatively regulate autophagy by promoting IP3 production via cAMP-Epac-Rap2B-PLC-ε pathway. Finally, IP3, via IP3R influence cytosolic Ca^2+^ levels, which can again activate calpains, thus creating a potential positive feedback loop for autophagy inhibition (Williams et al., 2008). Again, it is important to mention that although several different L-type calcium channel inhibitors as well as agonist were used in the study, the data showing the effect of siRNA mediated knockdown of L-type calcium channels and/or channel-dead mutants on autophagy are missing. It would be interesting as well to check the effect of these inhibitors on “negative control” cells lacking L-type calcium channels. In addition, verapamil is known to passively diffuse into the lysosome, where it becomes protonated and could cause an increase in lysosomal pH (Lemieux et al., 2004). This could lead to the inhibition of lysosome function and thus block fusion with the autophagosome.

Two-pore channels (TPC) have been also proposed to regulate autophagy (Pereira et al., 2011; Gomez-Suaga et al., 2012). Mammalian TPC family comprise two members TPC1 and TPC2, widely expressed in humans and localized intracellularly on endolysosomes, with TPC2 being specifically targeted to lysosomes. Several groups proposed TPC as a mediator of endolysosomal calcium release in response to the elevation of the second messenger, nicotinic acid adenine dinucleotide phosphate (NAADP) (Calcraft et al., 2009; Galione et al., 2009). Recently Pereira et al. demonstrated that NAADP stimulates autophagy via TPCs in rat astrocytes (Pereira et al., 2011). The authors showed that NAADP mediated increase in the number of LC3-GFP puncta was reduced in cells, transfected with dominant negative TPC2 L265P construct, suggesting the importance of TPC2 for autophagy (Pereira et al., 2011). TPC2 channel has been also proposed to be involved in LRRK2 induced autophagy (Gomez-Suaga et al., 2012).

The MCU that was recently identified as a channel responsible for mitochondrial Ca^2+^ uptake (Baughman et al., 2011; De Stefani et al., 2011) has been demonstrated to have the role in autophagy regulation. Indeed, Cardenas et al. showed that the uniporter inhibitor Ru360 inhibited cell O_2_ consumption rate, activated AMPK, and induced autophagy (Cardenas et al., 2010). In line with these data, MCUR1(mitochondrial calcium uniporter regulator 1) was shown to regulate autophagy (Mallilankaraman et al., 2012). MCUR1 represents an integral membrane protein that is required for MCU-dependent mitochondrial Ca^2+^ uptake. Knockdown of MCUR1 in HeLa and HEK293T cells reduced cell O_2_ consumption rate, activated AMPK, and induced macroautophagy (Mallilankaraman et al., 2012). Importantly, stable knockdown of MCU in HeLa cells elicited essentially the same effects, confirming the regulatory role for MCU toward autophagy (Mallilankaraman et al., 2012).

MPTP has been also suggested to be implicated in autophagy regulation. Elmore et al. proposed that mitochondrial permeability transition (MPT) initiates autophagy in rat hepatocytes. Although the mechanism by which the MPT signals autophagic sequestration was not investigated in this work, the authors hypothesized that factors released from the mitochondrial intermembrane space as a consequence of MPT could stimulate autophagy (Elmore et al., 2001). It is not clear if the Ca^2+^-release channel function of the MPTP is essential here as well. A functional MPTP was also shown to be required for starvation-induced mitochondrial autophagy (Carreira et al., 2010). The authors demonstrated that starvation induced mitochondrial depolarization in cardiac cells. This depolarization was prevented by cyclosporin A (MPT inhibitor). Further, the authors showed that cyclophilin D a component of the MPTP, is required for mitochondrial removal by starvation-induced autophagy. Interestingly, cardiomyocytes from cyclophilin D deficient mice failed to upregulate autophagy in response to nutrient deprivation, suggesting that MPTP is essential here (Carreira et al., 2010). Again the role of calcium and the importance of calcium permeability for MPTP in the regulation of autophagy were not assessed in this study.

## Potential new candidates for autophagy regulation in cancer treatment

Aside from ion channels, described above, all the other calcium permeable channels could potentially be involved in autophagy regulation, as they contribute to the changes in cytosolic calcium levels. Here, we will provide several hypotheses for autophagy regulation by some of the calcium permeable channels that have not been shown to be directly involved in autophagy regulation. Further, we will propose the possible link between calcium permeable channels, autophagy and cancer progression and therapeutic response. Considering both physiological roles as well as cellular localization we selected several calcium permeable channels, which in our opinion could have an impact on autophagy.

TRPML2 channel, a member of the mucolipin family, has been shown to localize to late and recycling endosomes as well as lysosomes (Zeevi et al., 2009; Cheng et al., 2010). Recent study claimed that TRPML2 does not appear to play a role in starvation-induced autophagy (Zeevi et al., 2010). However, TRPML2 knockdown was demonstrated to induce lysosomal inclusions accumulation in HEK cells (Zeevi et al., 2009). This fact along with the endolysosomal distribution of TRPML2 indicates the potential role of TRPML2 in the regulation of basal as well as other types of autophagy.

TRPM2 is known as a chanzyme, combining two functions: of an ion channel and an enzyme, since the C-terminal of TRPM2 contains enzymatically active adenosine diphosphoribose (ADPR) hydrolase domain (Sumoza-Toledo and Penner, 2011). TRPM2 has been shown to be activated and regulated by variety of stimuli including ADPR, H_2_O_2_, NAADP, pH, and cytosolic calcium. It is involved in numerous physiological processes, such as production of cytokines, insulin secretion, oxidative stress, apoptosis (Jiang et al., 2010). TRPM2 functions as a Ca^2+^-permeable channel on the cell surface, but recently TRPM2 has been shown to be also localized intracellularly on the late endosomal and lysosomal membranes where it functions as a lysosomal Ca^2+^ release channel (Lange et al., 2009). Thus, it can possibly affect autophagy in the same manner as TRPML and TPC channels. Interesting that both Ca^2+^-entry and Ca^2+^-release channel functions of TRPM2 were shown to be important in H_2_O_2_-induced beta-cell death (Lange et al., 2009). Further, H_2_O_2_ is a known activator of autophagy (Chen et al., 2008). Thus, potentially TRPM2 could be involved in H_2_O_2_-induced autophagy.

Another interesting candidate is the cold receptor TRPM8, which is found in sensory neurons, where it constitutes the principal detector of cold (<~28°C) (Bautista et al., 2007). In addition to its role as plasmalemmal Ca^2+^ channel, TRPM8 could function as intracellular Ca^2+^-release channel on the ER membrane (Zhang and Barritt, 2004; Thebault et al., 2005). Initially, TRPM8 was cloned from the human prostate as prostate-specific gene, which is upregulated in malignant tissues (Tsavaler et al., 2001). The role of TRPM8 in cancer was extensively studied in recent years, and published data suggest that TRPM8 could be involved in proliferation, differentiation and apoptosis in cancer cells (Zhang and Barritt, 2004; Thebault et al., 2005). Given the localization of TRPM8 on ER, it would be interesting to study the possible autophagy regulation by TRPM8-mediated Ca^2+^ release from the ER. The potential mechanisms could be the same as for IP3 receptor.

It is worth to note that TRPV1 channel, discussed above, has also been found to be expressed intracellularly at the ER and trans-Golgi network (Turner et al., 2003), so apart from the autophagy modulating mechanism provided above, TRPV1 could also be involved in another autophagy related signaling pathways.

TRPP2, the product of the gene mutated in autosomal dominant polycystic kidney disease (ADPKD), is another possible candidate for autophagy regulation. It is widely expressed, with a highest level in the kidney, and primarily localized in cilia, where it seems to function as a mechanosensor involved in the nodal ciliary movement (Delmas et al., 2004). TRPP2 might function as a plasma membrane calcium-permeable channel (when interacting with TRPP1) or as a calcium release channel located in the ER (Hanaoka et al., 2000; Cahalan, 2002; Koulen et al., 2002). Interesting, TRPP2 was shown to function as a calcium-activated intracellular calcium release channel—property reminiscent of IP3 receptors and ryanodine receptors (Koulen et al., 2002). Thus, potentially TRPP2 could regulate autophagy in a way similar to IP3R.

Interesting, although ryanodine receptor (RyR) constitutes the major cellular mediator of calcium-induced calcium release, the data on its role in autophagy is very scarce. As it was mentioned above, overexpression of ryanodine receptor type 2 in triple IP3R-deficient DT40 cells was without effect on constitutive autophagy (Cardenas et al., 2010). Despite this, we believe that more experiments are required to unravel the role of RyR in autophagy regulation.

The next candidate, TRPV2, is a Ca^2+^ permeable non-selective cationic channel, which has been found to be activated by noxious heat (>50°C), growth factors (i.e., IGF) and stretch (Caterina et al., 1999; Kanzaki et al., 1999; Muraki et al., 2003). It was shown that insulin induced translocation and insertion of TRPV2 into the plasma membrane in a PI3K-dependent manner (Aoyagi et al., 2010). Recent studies revealed the role for TRPV2 in promoting prostate cancer migration and progression to androgen resistance (Monet et al., 2010). Interestingly, Saito et al. demonstrated the function of 2-APB-activated and Rutenium Red-inhibited calcium-permeable ion channel in early endosomes (Saito et al., 2007). The authors reported that this channel has similar pharmacology to that of TRPV2. As early endosomes, and fusion of autophagosomes with functional early endosomes have been shown to be essential for autophagy (Razi et al., 2009), we hypothesize that TRPV2, which apparently forms early endosomal Ca^2+^-release channel, could be involved in autophagy where it may regulate fusion between autophagosomes and early endosomes. Function on the plasma membrane as well as dependence on PI3K suggest possible complex role in autophagy regulation.

ORAI1 (the calcium release-activated calcium channel protein 1) which constitutes a major molecular component of store-operated calcium (SOC) channels (Hewavitharana et al., 2007) also represents an attractive candidate for autophagy regulation. Recently, Abdelmohsen et al. reported that microRNA miR-519 stimulates autophagy through the downregulation of ORAI1 and ATP2C1 proteins, increase in the cytosolic Ca^2+^ levels, activation of Ca^2+^-activated calmodulin kinase II (CaMKII) as well as glycogen synthase kinase 3β (GSK3 β) and subsequent p21 upregulation (Abdelmohsen et al., 2012). However, the direct link between autophagy stimulation and functionality of ORAI1 as a Ca^2+^ permeable channel is missing. Thus, additional experiments using specific agonists and antagonists as well as siRNA knockdown of ORAI1 are needed to confirm its role in autophagy regulation.

**Figure 1:**
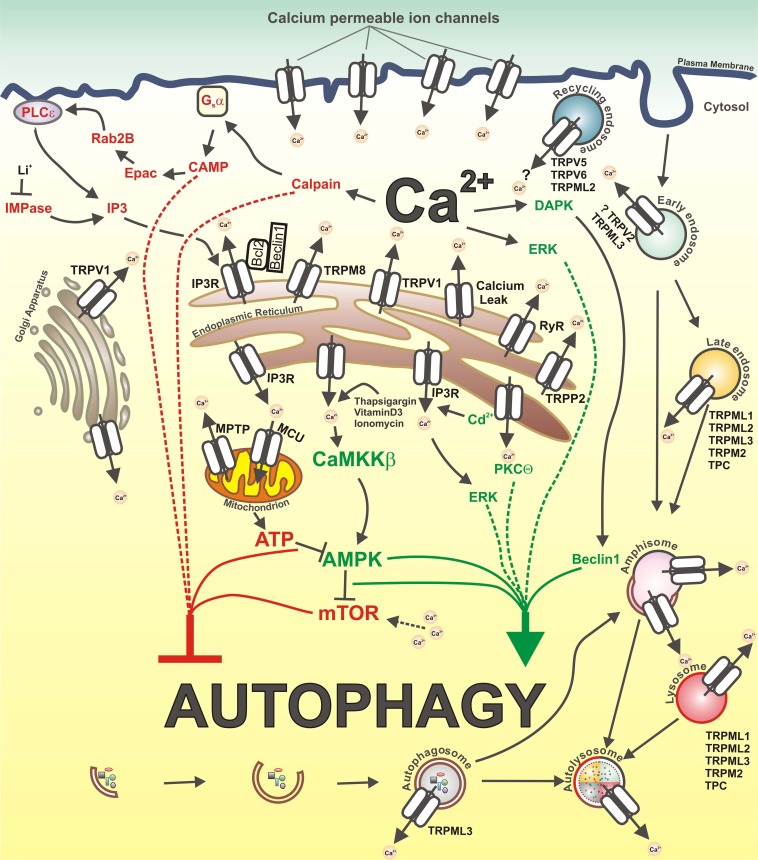
**Calcium and calcium-permeable channels in the control of autophagy.** Inhibitory and stimulatory actions of Ca^2+^ on autophagy as well as calcium-permeable channels that could be potentially involved in autophagy regulation are depicted. Ca^2+^ mobilizing agents, such as vitamin D3, thapsigargin and ionomycin, lead to increase in cytosolic Ca^2+^ ([Ca^2+^]_cyt_) concentration and subsequent activation of CAMKK-beta, followed by AMPK-dependent mTOR inhibition and autophagy stimulation. ER-stress induced elevation of [Ca^2+^]_cyt_ levels activate PKCtheta which stimulates autophagy. Additionally, increased [Ca^2+^]_cyt_ induce activation of DAPK, which phosphorylates Beclin1, thereby promoting its dissociation from Bcl-X_L_ and Bcl-2 inhibitory proteins, and thus stimulate autophagy. Cadmium induces autophagy through elevation of cytosolic Ca^2+^ via IP3R and subsequent ERK activation. In contrast, constitutive IP3R mediated Ca^2+^ release to mitochondria maintains ATP production and AMPK inhibition, thereby suppressing autophagy. The inhibition of IMPase by Li^+^ causes a decrease in IP3 levels and autophagy induction. Further, IP3R-dependent Ca^2+^ release from the ER as well as amino acids-induced increase in [Ca^2+^]_cyt_ maintain an elevated mTORC1 activity, thus inhibiting autophagy. In addition, IP3R represses autophagy through Bcl-2-mediated binding of Beclin1. Also, increased Ca^2+^ influx through L-type calcium channels on the plasma membrane activates calpains and consequently Gsalpha/Adenylyl cyclase/cAMP/Epac/Rap2B/PLC-ε pathway which negatively regulates autophagy by promoting IP3 production, IP3R activation and Ca^2+^ release.

A subset of other channels, found to be localized both on plasma membrane and intracellular vesicles (i.e., recycling endosomes), including TRPC3, TRPV6, and TRPV5, could also potentially modulate autophagy, although at present it's not clear if these channels are functional intracellularly (Dong et al., 2010; Toro et al., 2011). A graphic overview of the calcium-related mechanisms of autophagy regulation is presented in Figure 1.

Thus, available data strongly suggests that calcium permeable channels represent good candidates for autophagy regulation. Given that both autophagy and calcium-permeable ion channels have a role in cancer, this can be highly valuable in order to achieve specific outcomes in anti-cancer therapy. Ion channels could provide some advantages, when targeting autophagy *in vivo* for cancer treatment. Indeed, most ion channels are localized on cell surface, thus they can be subjected to antibody-based targeting that can be particularly useful in the case of channel upregulation in cancer. Moreover, anti-channel antibodies could be used as carriers for radionuclides, toxic molecules or nanoparticles, which can themselves affect autophagy and as such influence cell fate.

The growing number of studies pointing on the fact that inflammation increases the incidence of cancer (Mantovani et al., 2008). Autophagy has been linked to both cancer and inflammation, and is often defective in the inflammatory conditions (White et al., 2010). Among them, Crohn's disease and pancreatitis have been associated with an increased risk of colorectal and pancreatic cancers, respectively (Freeman, 2008; Raimondi et al., 2010). Accordingly, these pathological states are characterized by the accumulation of damaged organelles and poly-ubiquitinilated protein aggregates, ROS production and DNA damage, the factors that create a cancer-promoting environment (Gukovsky et al., 2012; Nguyen et al., 2013). Hence, this implies that functional autophagy stimulation, to eliminate dangerous garbage, may constitute an effective approach to cancer prevention. Interestingly, a number of calcium-permeable ion channels, including TRPV1, TRPV4, TRPA1, and TRPM8, were shown to be regulated by inflammatory mediators (Nilius et al., 2007; Kochukov et al., 2009; Zhang et al., 2012). Thus, these channels can represent potential targets to stimulate autophagy in inflammatory conditions in order to avert tumorigenesis initiation. Additionally, lysosomal dysfunctions have been reported in pancreatitis (Gukovsky et al., 2012), therefore it could be interesting to consider lysosomal ion channels as well.

On the other hand, in existing tumors autophagy may favor survival and progression. Thus, the possible anticancer therapy should be focused on autophagy inhibition. In this case, considering calcium-permeable channels as a potential tool to target autophagy could also be useful. More specifically, as we discussed above, a number of calcium-permeable ion channels exhibit altered expression in cancer cells. For instance, TRPM8 is upregulated in androgen-dependent prostate cancer cells (Zhang and Barritt, 2004; Thebault et al., 2005). This could possibly influence intracellular calcium levels and consequently autophagy. Hence, targeting TRPM8 as well as another channels overexpressed in cancers could provide an additional control over autophagy, particularly during chemotherapy, and as such contribute to cancer treatment.

## Conclusions

Calcium-permeable ion channels have emerged as important regulators of autophagy and the effect of such regulation most likely depends on Ca^2+^ signals in a spatially restricted subcellular domains. Apparently, such regulation can represent a fundamental mechanism of fine tuning the autophagy. However, the data concerning this subject is very limited, thus further studies are needed to understand the variety of mechanisms, by which calcium channels can influence autophagy.

Accumulated data proves that both calcium-permeable ion channels and autophagy are implicated in cancer initiation and progression as well as chemotherapy resistance. Paradoxically, autophagy has opposite roles in cancer, with both tumorigenesis suppressor action, in particular at the early stages of tumor initiation and cancer promotion effect resulting in tumor cell survival, chemotherapy resistance and cancer progression. Thus, it is important to unravel autophagy regulating pathways to most effectively target autophagy to cure cancer. Identification of the connections between calcium channels and autophagy could define a new strategy in cancer treatment, and identify useful tools and biomarkers for the elaboration of effective anti-cancer therapies. Moreover, as malfunction of autophagy has been linked to a wide range of human pathologies including liver disease, neurodegeneration, Crohn's disease and cancer, uncovering novel mechanisms of autophagy regulation by calcium permeable ion channels could have a broad impact on the “Autophagy” field and contribute to the developing of autophagy as a potential clinical approach to cure diseases.

### Conflict of interest statement

The authors declare that the research was conducted in the absence of any commercial or financial relationships that could be construed as a potential conflict of interest.
